# Amidated and Aminated PMSSO-Hydrogels as a Promising Enzyme-Sensitive Vehicle for Antianemic Drugs

**DOI:** 10.3390/gels11020118

**Published:** 2025-02-06

**Authors:** Polina Orlova, Ivan Meshkov, Sergei Sharikov, Vsevolod Frolov, Anna Skuredina, Pavel Markov, Zoya Bobyleva, Grigorii Lakienko, Egor Latipov, Ilya Kolmogorov, Sergey Vasiliev, Alexandra Kalinina, Aziz Muzafarov, Irina Le-Deygen

**Affiliations:** 1 Department of Chemistry, Lomonosov Moscow State University, Moscow 119991, Russia; p.orlova2021@mail.ru (P.O.); sharikovsv@my.msu.ru (S.S.); frolov_vsevolod@mail.ru (V.F.); anna.skuredina@yandex.ru (A.S.); markovpo@rambler.ru (P.M.); zoyamostovik@gmail.com (Z.B.); bojk25@gmail.com (G.L.); kolmogorov2001@mail.ru (I.K.); 2 Enikolopov Institute of Synthetic Polymeric Materials, Russian Academy of Sciences (ISPM RAS), Moscow 117393, Russia; ivanbm@ispm.ru (I.M.); kalinina@ispm.ru (A.K.); aziz@ispm.ru (A.M.); 3 Institute of Nanotechnology of Microelectronics, Russian Academy of Sciences (INM RAS), Moscow 119334, Russia; la_019@mail.ru; 4 Federal Research Center of Problems of Chemical Physics and Medicinal Chemistry RAS (FRC PCP MC RAS), Moscow 142432, Russia; viesssw@mail.ru

**Keywords:** anemia, cyclodextrin, hydrogels, drug delivery, polymethylsilsesquioxane

## Abstract

In this study, we report the synthesis and characterization of aminated poly(methyl silsesquioxane)-based hydrogels ((AP/MS)SO-hydrogels) as potential enzyme-sensitive vehicles for antianemic drugs. The hydrogels were synthesized via sol–gel polymerization and functionalized with amine groups. Characterization techniques included Congo red assay, Brunauer–Emmett–Teller (BET) surface area analysis, scanning electron microscopy, elemental analysis, ^13^C NMR, ^29^Si NMR, and ATR-FTIR spectroscopy and microscopy of hydrogels. The sorption of ferric chloride and ferrous D-gluconate, as well as complexes of ferrous D-gluconate with HPCD, was evaluated. Crosslinking of the gel with bifunctional agents was performed to create a new amide enzyme-sensitive bond, followed by infrared characterization of the crosslinked product. Trypsin-mediated degradation studies demonstrated the sensitivity of the hydrogel to enzymatic cleavage under model conditions. Iron release experiments in gastric and intestine-simulating media confirmed prolonged release. Overall, our findings suggest that aminated PMSSO-hydrogels hold promise as versatile and biocompatible carriers for targeted delivery of antianemic agents, warranting further exploration in preclinical and clinical applications.

## 1. Introduction

Anemia constitutes a worldwide health hazard, impacting millions, particularly children and women of reproductive age [1]. Intravenous iron forms are utilized in life-threatening situations; nevertheless, oral medication is generally preferred in most instances. Iron is absorbed by the mucous membrane of the duodenum; however, usually drug release occurs in the stomach due to the harsh digestive conditions [2], resulting in significant adverse effects that diminish patients’ quality of life and compromise therapeutic efficacy. Consequently, the administration of oral iron supplements frequently results in adverse consequences due to the premature release of iron from the formulation.

Hydrogels are a perspective drug delivery system for the peroral delivery of iron compounds. Both natural [3] and synthetic [4] hydrogels are applied. Hydrogels have significant advantages: high sorption capacity, stability during storage, biocompatibility, and often biodegradability. We recently demonstrated that organosilicon poly(methyl silsesquioxane)-based (PMSSO) hydrogels can partially retard the release of iron in the stomach; nonetheless, these systems require enhancement [5,6]. Regulation of PMSSO-hydrogel synthesis conditions allows for obtaining porous structures with specified characteristics: high adsorption capacity and a high degree of selectivity with respect to various substances, including biologically active molecules and pharmaceuticals.

Research is currently focused on examining the specific characteristics of intestinal function to develop selective-release drug delivery systems [7]. The intestine functions as a distinct system comprising both human and abundant bacterial cells from the microbiome. Notably, it exhibits high enzymatic activity from pancreatic enzymes and intestinal juice, a slightly alkaline pH, and active peristalsis, which promotes the swift transit of substances [8,9]. The proposed methods are unified by the selection of multipoint complexes for the stability of delivery systems; however, it is preferable to incorporate additional parameters that govern the transit through the stomach without compromising the target drug. To do this, it is recommended to utilize “staples”, which are chemical linkages formed between the bifunctional agent and the functional groups on the carrier gel’s surface. The choice of the chemical composition of the “staple” can facilitate targeted hydrolysis in the intended organ.

Thus, a possible strategy to promote selective drug release in the intestines involves the formation of enzyme-sensitive crosslinks on the gel surface, which can further impede the release of contents through an additional phase of enzymolysis [10]. A recent review demonstrates methods for employing polysaccharides in enzyme-sensitive intestine delivery systems [11]. The intestine has high activity of digestive proteases and lipases, which can be used to implement an enzyme-sensitive strategy. Despite the high activity of pepsin in the stomach, the use of protease-sensitive systems has proven itself [12]. Trypsin, chymotrypsin, and elastase are key intestinal proteases, but other enzymes, in particular enzymes of the intestinal microbiota, are also active in the intestine. Research indicates potential for employing trypsin-sensitive zones in hydrogels in biomedical applications [13].

The creation of organosilicon hydrogels with sensitivity to digestive enzymes would expand the scope of application and open up new prospects for the use of such materials in biomedicine. The possibilities of organosilicon synthesis in combination with sensitivity to biological stimuli (such as the presence of enzymes) could potentially be the basis for a new class of drug carriers.

The aim of this work is to develop a new class of enzyme-sensitive PMSSO hydrogels with introduced amide crosslinks, sensitive to intestinal proteases, for oral delivery of iron compounds. For this purpose, we aimed to synthesize aminated PMSSO hydrogels ((AP/M)SSO) and then introduced amide crosslinks onto their surface using adipic acid. Two synthesis strategies were selected: synthesis using adipoyl dichloride and the carbodiimide method.

## 2. Results and Discussion

### 2.1. Synthesis and Characterization of Aminated PMSSO Hydrogels ((AP/M)SSO)

**Scheme 1:**
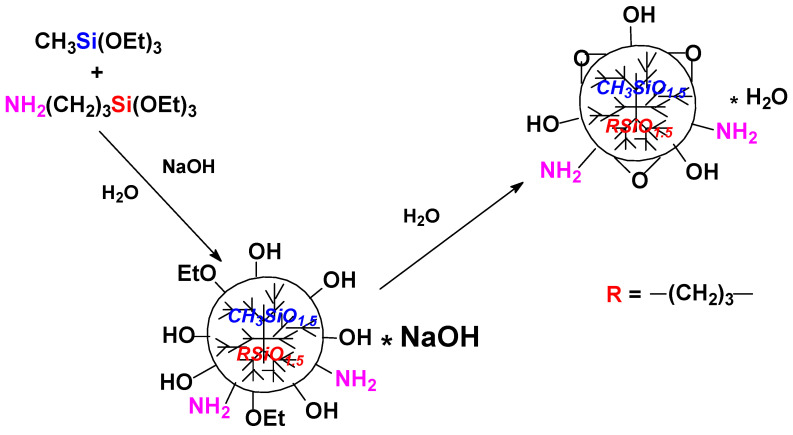
Hybrid polyaminopropylmethylsilsesquioxane ((AP/M)SSO) hydrogel synthesis.

The synthesis of PMSSO hydrogels is usually conducted via an eco-friendly method previously developed in ISPM RAS [14], which is based on the hydrolytic polycondensation of alkoxysilane. The synthesis of polymethylsilsesquioxane (PMSSO) hydrogels from methyltriethoxysilane involves a two-stage process: hydrolysis and heterofunctional condensation of intermediates, resulting in PMSSO sol, followed by the conversion of polyhydroxyl oligomers into a hydrogel through homofunctional condensation. The initial phase involves utilizing specific quantities of alkali required for the synthesis of a soluble sodium methylsiliconate sol, akin to the method employed for producing hydrophobizing agents. The sol–gel transition, akin to hydrophobization, transpires when the negative charge of oxygen in hydroxyl groups, solvated by aqueous alkali, is neutralized, hence inhibiting condensation. The second stage also encompasses the maturity of the gel, which involves the ultimate stabilization of the structure through a network of hydrogen bonds. In this paper, we have for the first time employed this method to produce (AP/M)SSO hydrogels—PMSSO-hydrogels modified with amino groups. An amino-containing 3-aminopropyl)triethoxysilane (APTES) is introduced in the initial step according to Scheme 1.

We altered the mole fraction of APTES to produce hybrid hydrogels with varying amino group concentrations. We employed alkali and ethanol as additives to stabilize the gel (Table 1). Four samples of hybrid hydrogels were synthesized based on varying proportions of additives and APTEOS, which were subsequently analyzed using BET and NMR techniques, with sorption capacity assessed by the Congo red test (Table 1). ^13^C and ^29^Si NMR indicated the formation of a silsesquioxane hydrogel network (Figure S1) as well as the presence of NH_2_ groups.

To verify the presence of nitrogen in the synthesized gels, we conducted elemental analysis to assess the concentrations of the main elements: silicon, carbon, nitrogen, and hydrogen. According to the results presented in Table 2, a higher molar % of APTES indeed leads to a higher content of nitrogen in final hydrogels.

**Figure 1:**
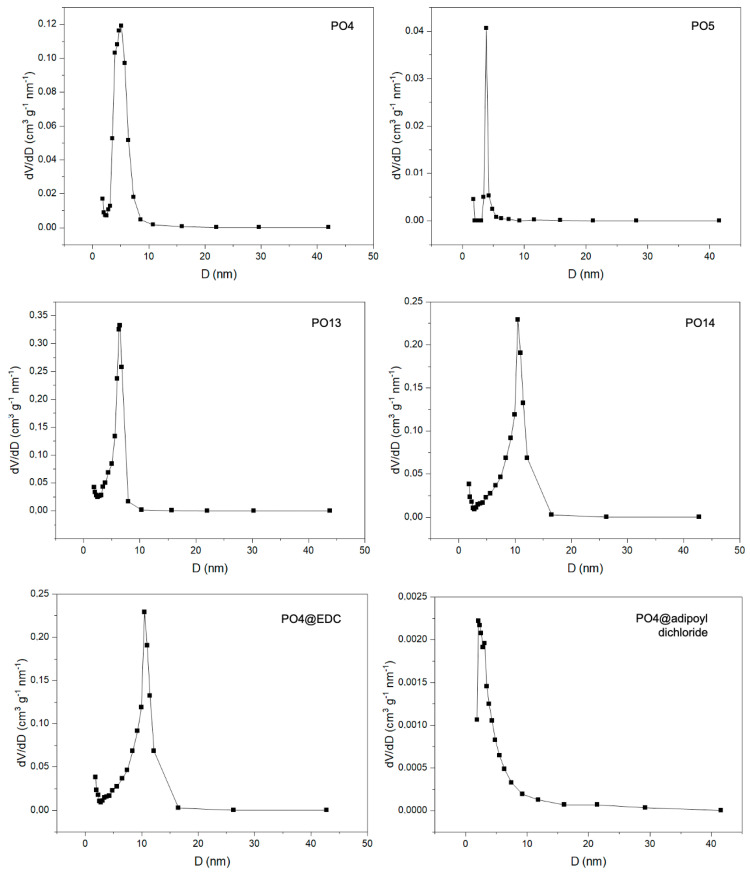
Pore size distribution of (AP/M)SSO) hydrogel samples according to the BET analysis.

BET analysis confirmed the formation of a porous structure (Figure 1). Figure S2 represents N_2_ adsorption–desorption isotherms of hydrogels. Except for the PO5 sample, (AP/M)SSO hydrogels possess high surface with pore volumes of 0.3–0.8 mL/g. All samples possess fine pore size distribution with mean pore size 5–10 nm. A higher amount of APTES during synthesis provides (AP/M)SSO hydrogels with higher sorption capacity in the Congo red test. Interestingly, compared to the PMSSO hydrogels without amino groups, sorption capacity increases dramatically: for PMSSO hydrogel, it was less than 5 micromole/g [6]. This difference could be caused by a strong interaction between SO_3_^−^ groups in Congo red molecule and amino groups in (AP/M)SSO hydrogels.

**Figure 2:**
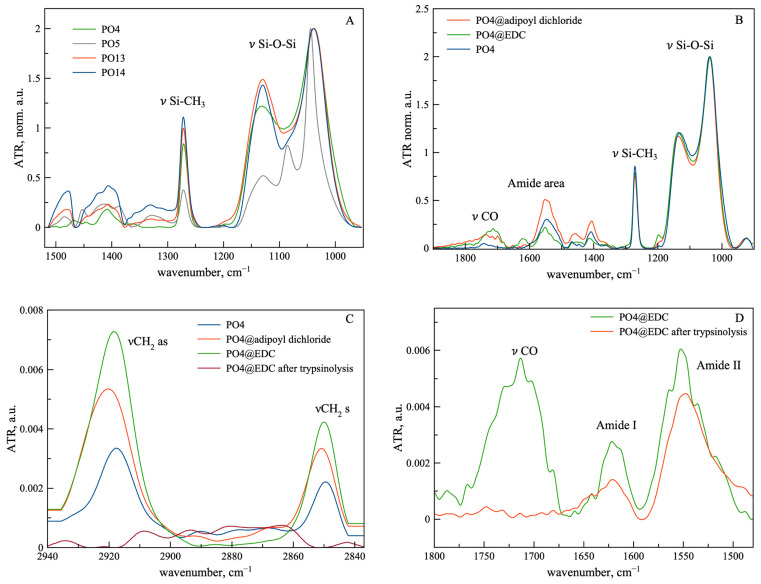
ATR-FTIR spectra of (AP/M)SSO hydrogels. (**A**) Min–max normalized spectra of hydrogels PO4 (green line), PO5 (grey line), PO13 (red line), PO14 (blue line). (**B**) Min–max normalized spectra of hydrogels PO4 (black line), PO4@adipoyl dichloride (red line), PO4@EDC (green line). (**C**) Spectral area of CH_2_ valence oscillation of hydrogels PO4 (blue line), PO4@adipoyl dichloride (red line), PO4@EDC (green line), PO4@EDC after 30 min of trypsinolisis (bordo line). (**D**) Amide spectral area of hydrogels PO4@EDC (green line) and PO4@EDC after 30 min of trypsinolisis (red line). Aqueous media, pH 7.0, 22 °C.

ATR-FTIR spectroscopy was utilized to analyze the fine structure of synthesized hydrogels. Figure 2A presents the spectra with a consistent pattern of organosilicon hydrogels. The most pronounced absorption bands correspond to the valence vibrations of bonds ν Si-CH_3_ (1270 cm^−1^) and ν Si-O-Si (1200–100 cm^−1^ area, two bands). The spectral pattern of the PO5 sample, distinct from all others, is noteworthy: the presence of a third absorption maximum in the 1200–1000 cm^−1^ range signifies the development of additional covalent bonds. This conclusion aligns well with the BET data, which indicates that PO5 exhibits the lowest porosity, but the other three samples possess comparable features.

**Figure 3:**
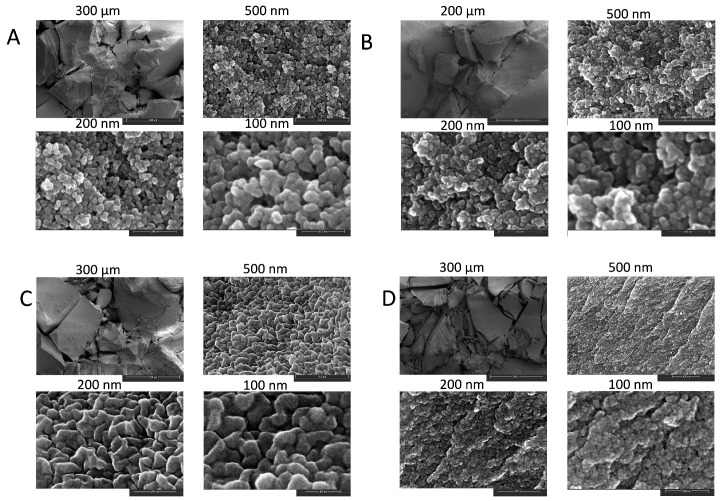
Scanning electron microscopy of PO4 (**A**), PO4@EDC (**B**), PO4@adipoyl dichloride (**C**) and PO5 (**D**) hydrogels with various magnifications.

The surface morphology of the produced hydrogels was studied using samples PO4 and PO5 as examples. Figure 3 presents pictures acquired by scanning electron microscopy at various magnifications. Sample PO4 exhibits a more advanced surface with a distinctive “curly” morphology, whereas sample PO5, which according to BET data possesses a considerably less developed surface area, displays a less intricate surface.

The aminated hydrogel sample selected for further modification must possess a well-developed porous surface and a high concentration of amino groups. Elemental analysis indicates that samples PO13 and PO14 possess a reduced nitrogen content, while sample PO5, exhibiting a nitrogen content similar to PO4, demonstrated inadequate porosity as per BET data. Consequently, sample PO4 was chosen for more investigations.

### 2.2. ((AP/M)SSO) Hydrogels as Carriers for Ferric Chloride and Ferrous D-Gluconate

Considering the obtained hydrogels as potential carriers for antianemic drugs, it is vital to evaluate its sorption capacity for not only the model dye Congo red, but also iron compounds. Table 3 presents the results on the sorption efficiency for two iron salts—ferric chloride FeCl_3_ and ferrous D-gluconate. All hydrogels under consideration demonstrated high sorption capacity for ferric chloride (50–60%), while for ferrous D-gluconate which is considered as an important antianemic therapeutics, the results vary. PO13 and PO14 hydrogels sorption efficacy dropped dramatically, while for PO4 and PO5 it was less pronounced. To overcome this issue, we employed a method involving the synthesis of inclusion complexes of iron D-gluconate with 2-hydroxypropyl-beta-cyclodextrin (HPCD) for the more promising gels PO4 and PO5. This strategy previously allowed us to increase the sorption capacity for PMSSO hydrogels [6].

Complexes ferrous D-gluconate–HPCD with synthetized by the kneading technique and characterized according to the previously published protocol. Complex formation was proved by ATR-FTIR spectroscopy (Figure S3). For the spectrum of ferrous D-gluconate, the following main bands were observed: 1593 cm^−1^, vibrational vibrations of ν COO-; 1360–1415 cm^−1^, corresponding to δ OH-, and 1050–1130 cm^−1^, corresponding to ν C-O. For the spectrum of HPCD, 1360–1415 cm^−1^, corresponding to δ OH-, and 1050–1130 cm^−1^, corresponding to vibrations of ν C-O, were also observed. As a result of the formation of the complex, these bands were also observed, but the intensity of the band at 1593 cm^−1^ (ν COO-) decreased after the addition of HPCD compared to the spectrum of 0.04 M iron D-gluconate. It also follows from the data obtained that the intensity of 1050–1130 cm^−1^ (ν C-O) increases upon complex formation. Thus, we can assume that complexation occurs as a result of binding between COO- of iron D-gluconate and OH- of HPCD, as well as OH- of both compounds.

The formation of the complex allowed the sorption capacity of PO4 and PO5 hydrogels to increase almost threefold. Thus, for further practical use of (AP/M)SSO hydrogels, one should consider them as cargo complexes with HPCD. On the other hand, the introduction of amino groups into organosilicon gels is the first step prior to the formation of extra enzyme-sensitive crosslinks. Sample PO4 was selected for this synthesis due to its greater number of amino groups on one side, along with a well-developed surface and significant porosity on the other side.

### 2.3. Synthesis and Characterization of Amidated PMSSO Hydrogels ((AP/M)SSO)

The approach of modifying the structural–functional properties of hydrogels through the synthesis of extra amide bonds appears promising [15]. Bifunctional compounds are frequently employed as crosslinking agents, particularly derivatives of dicarboxylic acids such as succinic [16] or adipic acid [17]. In this work, we chose adipic acid to form longer staples on the gel surface, potentially more sensitive to trypsinolysis [18].

**Scheme 2:**
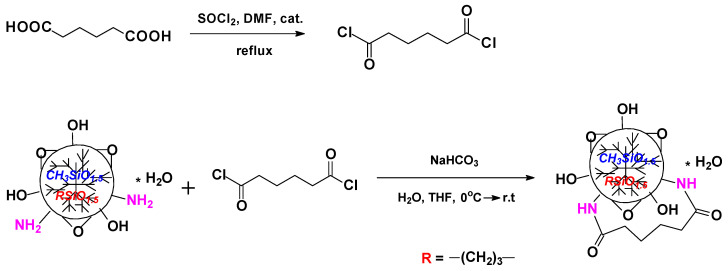
Hybrid polyaminopropylmethylsilsesquioxane ((AP/M)SSO) with additional crosslinks hydrogel synthesis via adipoyl dichloride way.

Two approaches were employed to create additional crosslinks in hydrogels. The first strategy utilizes adipoyl dichloride, a well-studied technique to obtain amides (Scheme 2). Several papers [19,20] describe this way to synthetize amide bonds in gels, so here we applied this way as well.

**Scheme 3:**
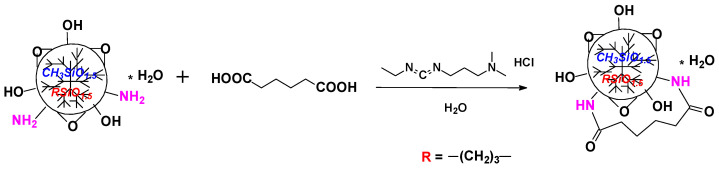
Hybrid polyaminopropylmethylsilsesquioxane ((AP/M)SSO) with additional crosslinks hydrogel synthesis via carbodiimide way.

The second strategy (Scheme 3) entailed synthesis, starting with the activation of adipic acid using EDC carbodiimide. Carbodiimide synthesis has established itself as the gold standard for amide synthesis, especially when using unsymmetrical carbodiimides such as EDC [21,22].

The physicochemical characterization of the synthetized amidated gels demonstrated the gap between the two synthesis methodologies. The sample PO4@EDC exhibited well-developed porosity, with the specific surface area of the pores slightly exceeding that of the original sample PO4, although the D90 value decreased (Table 1). This signifies the development of supplementary segments without considerable disruption to the hydrogel structure. The hypothesis was validated by scanning electron microscopy data (Figure 3): the “curly” morphology of the sample surfaces was consistent across all magnifications. A different pattern was identified for the sample PO4@adipoyl dichloride. The specific surface area based on BET data decreased by over an order of magnitude, whereas the D90 parameter conversely increased (Table 1). This signifies substantial alterations in the hydrogel structure, confirmed by SEM data (Figure 3): variations in surface morphology were evident even with a minor magnification. This may be attributed to the occurrence of side processes during the reaction outlined in Scheme 2, resulting from more aggressive synthesis conditions. For biological applications, carriers synthesized under mild conditions with controlled processes are preferable. Thus, the carbodiimide way could be considered preferable.

The structure of the synthesized gels was confirmed by ATR-FTIR spectroscopy. Figure 2B illustrates the spectra of the control hydrogel PO4 alongside the obtained crosslinked gels, produced by adipoyl dichloride synthesis (PO4@adipoyl dichloride) and via carbodiimide synthesis (PO4@EDC). Conservative spectral patterns in the area of ν Si-CH_3_ (1270 cm^−1^) and ν Si-O-Si (1200–100 cm^−1^) bands indicate that the organosilicon network of the gel maintains its structure. Conversely, distinctive peaks emerge in the region associated with the absorption of the amide bond. Consequently, the Amide I and Amide II bands arise in the 1650–1500 cm^−1^ area, alongside a band corresponding to the valence vibrations of the C=O bond (1730 cm^−1^). It is likely that ester crosslinks are produced during the synthesis stage, which may also undergo enzymatic cleavage.

Since adipic acid contains a (CH_2_)_4_ fragment, absorption corresponding to the stretching vibrations of methylene groups should be expected in the ATR-IR spectrum of crosslinked hydrogels. In the spectrum of the initial PO4 hydrogel, absorption in this region is associated with the presence of ethyl substituents in the structure (Scheme 1). The formation of crosslinks with adipic acid leads to a noticeable increase in absorption in the region characteristic of symmetric and asymmetric stretching vibrations of CH_2_ groups (Figure 2C). This effect is most pronounced for the sample obtained by the carbodiimide method, which indicates the creation of a denser network of crosslinks.

ATR-FTIR spectroscopy yields comprehensive data from the entire sample, whereas the sample’s surface topology is also important when it comes to crosslinking. To acquire further insights into this process, we employed IR microscopy, wherein the dried hydrogel sample is analyzed based on the intensity of specific absorption bands.

**Figure 4:**
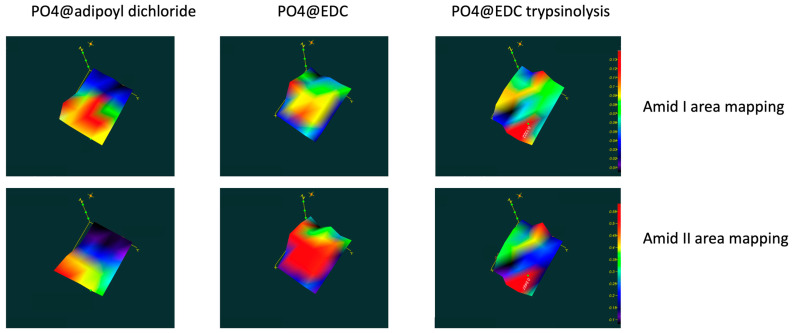
FTIR mapping for Amide I and Amide II areas for samples of dried (AP/M)SSO hydrogels: PO4@adipoyl dichloride, PO4@EDC, PO4@EDC after 30 min of trypsinolysis. Red coloring corresponds to the high intensity of the band, blue—low intensity. Mapping area was 1 × 1 cm, 22 °C.

The crosslinked hydrogel produced via acid chloride synthesis (PO4@adipoyl dichloride) has a less uniform distribution of amide bonds on the surface, as shown by the “focal” maxima observed in the Amide I and Amide II maps (Figure 4). Conversely, the sample PO4@EDC from carbodiimide synthesis has a more homogeneous distribution of amide bonds on its surface. The acquired data align with the findings of ATR-FTIR spectroscopy, supporting the hypothesis that carbodiimide synthesis facilitates the production of more densely crosslinked hydrogels. Mapping additionally disclosed a noteworthy detail: the strength of the Amide II band across the full surface of the sample map exceeds that of the Amide I band. This pattern is indicative of samples with a distinct orientation of amide bonds and is observed in the IR spectra of proteins, particularly those integrated into supramolecular assemblies [23]. Thus, carbodiimide synthesis produces more densely crosslinked gels than single-step synthesis with adipoyl dichloride.

The density of crosslinks also plays a key role in the sorption capacity of hydrogels. On the one hand, dense crosslinking can ensure the formation of cells for more effective cargo inclusion. On the other hand, too dense networks can be poorly included in the volume, and sorption will occur only on the surface. We determined how crosslinks affect the sorption capacity for iron d-gluconate in comparison with PO4 hydrogel (Table 3. It turned out that amide crosslinks significantly (3–5 times) increase the sorption capacity.

The increase in sorption capacity may be related to the formation of additional hydrogen bonds between the atoms in the amide bond and the sugar backbone of gluconate. The formation of hydrogen bonds has been previously described in the literature [24,25].

Thus, for the first time, we obtained amidated hydrogels with a variable degree of crosslinking, suitable for ferrous D-gluconate sorption. Next, we set the task of determining how a hydrogel with the densest degree of crosslinking would behave in the presence of protease.

### 2.4. Trypsinolysis

When developing oral formulations, it is crucial to examine how they react under enzymolytic conditions in the gastrointestinal system. Notwithstanding the complexity of this process, trypsin—a serine protease with supplementary esterase activity—is frequently employed as the model enzyme for this study. Trypsin exhibits quite wide substrate specificity, while its native substrates are peptides that contain lysine or arginine.

We performed a model experiment wherein hydrogel samples PO4 (control) and PO4@EDC as one with denser crosslinking were treated with trypsin for varying time intervals, thereafter analyzed using ATR-FTIR spectroscopy (from native samples) and IR microscopy (from dried samples). Given that trypsin exhibits both protease and esterase activity, alterations in the absorption range of 1800–1400 cm^−1^, which encompasses the distinctive absorption bands of these functional groups, should be expected.

Indeed, already 30 min after the start of trypsinolysis, significant changes are observed in this absorption region (Figure 2D). The intensity of the absorption bands of Amide I and Amide II decreases, indicating the degradation of amide bonds. Moreover, the absorption band of the stretching vibrations of the carbonyl group disappears, which corresponds to the esterase activity of trypsin.

Additional data can be obtained from the analysis of the absorption region of the stretching vibrations of methylene groups (Figure 2C). After 30 min of trypsinolysis, these absorption bands are not observed in the spectrum, indicating the removal of adipic acid fragments from the hydrogel network.

To obtain more detailed information about the surface topology of crosslinked hydrogel samples before and after trypsinolysis, we applied IR microscopy (Figure 4). We concentrated on the degradation of amide bonds and compared maps corresponding to the original PO4@EDC sample and the sample subjected to 90 min of trypsinolysis, utilizing the absorption bands Amide I and Amide II.

### 2.5. Iron Release Kinetics

**Scheme 4:**
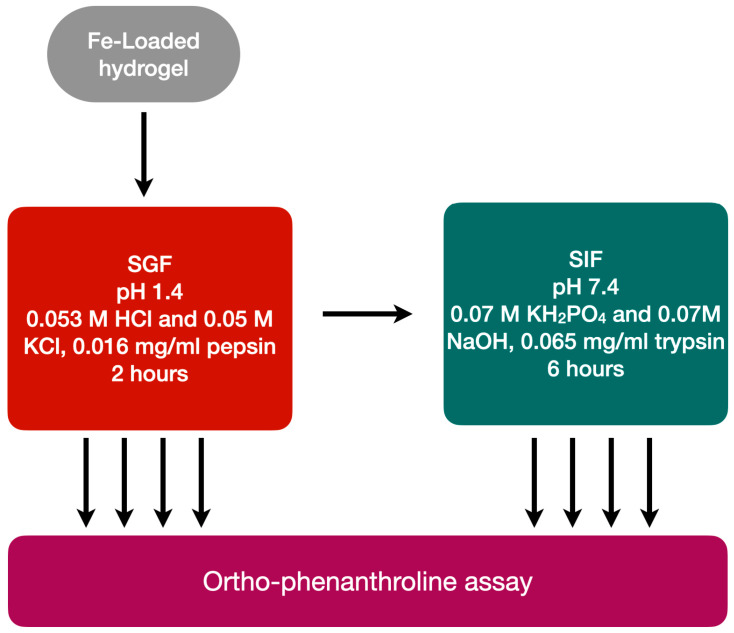
Pipeline of Fe-release experiment.

Drug-release kinetics play a crucial role in ensuring optimal pharmacokinetic parameters. Here we studied the kinetics of iron release in media, simulating the gastrointestinal tract. In order to study the role of amide crosslinking in the release kinetics we used SGF (simulated gastric media) and SIF (simulated intestinal media) containing main digestive proteases—pepsin and trypsin, correspondingly. The main pipeline of the experiment is presented in Scheme 4.

Figure 4 represents iron-release curves for samples PO4 and PO4@EDC.

**Figure 5:**
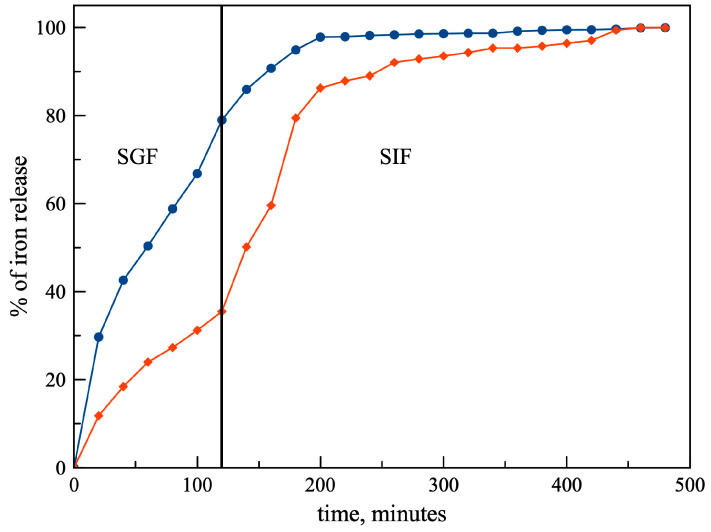
Iron release curves from PO4 hydrogel (blue curve) and PO4@EDC hydrogel (red curve). First 120 min of the experiment correspond to the SGF media, containing 0.016 mg/mL pepsin, pH 1.4 followed by 400 min corresponds to the SIF media, containing 0.065 mg/mL trypsin, pH 7.4. SIF and SGF media were prepared according to the USP. 37 °C. Black line marks the change from SGF to SIF.

During the first 120 min of the experiment, the samples were placed in the SGF media with pepsin, simulating gastric conditions (Figure 5). Throughout this period, the PO4 hydrogel released around 80 percent of its contents. In comparison to non-aminated PMSSO hydrogels, this exhibits a slower release; previously, we demonstrated that iron was entirely released from PMSSO hydrogels in approximately 100 min [6]. The hydrogel with amide crosslinks PO4@EDC exhibits a marked slowing down in release throughout the SGF. This process decelerates by more than twofold, indicating the crucial role of amide crosslinks in preventing premature release. Simultaneously, upon reaching the intestinal environment, which contains trypsin, the release significantly accelerates, and by the end of the fourth hour of the experiment, nearly all the iron is released.

Thus, for the first time, it was possible to create a new type of enzyme-sensitive amidated gels with a variable degree of crosslinking based on PMSSO hydrogels. The data obtained open up prospects for the creation of oral delivery systems with specified properties and controlled release of contents.

## 3. Conclusions

The development of oral delivery systems for low-molecular pharmaceuticals implies several features. The carrier must be biocompatible, biodegradable, possess high sorption capacity, and exhibit sensitivity to environmental factors, such as digestive enzymes. Organosilicon hydrogels, like PMSSO, have demonstrated efficacy as carriers; nevertheless, they lack sensitivity to enzymes. We attempted to address this issue by including amino groups during the polymer synthesis. Aminated hydrogels exhibit well-developed surfaces with nanosized pores and have the capability to absorb iron compounds, specifically ferric chloride (up to 69%) and less for ferrous D-gluconate (up to 13%). The sorption capacity can be enhanced by complexing ferrous D-gluconate with HPCD (up to 40%).

To create enzyme-sensitive amide crosslinks, we implemented two strategies: synthesis via adipoyl dichloride and via carbodiimide synthesis. The resulting hydrogels were characterized using spectral and microscopic methods, and the formation of amide crosslinks was confirmed. The hydrogel PO4@EDC synthesized by the carbodiimide way had a similar “curly” morphology compared to the control PO4 and close porosity values. The adipoyl dichloride way leads to a significant change in the surface morphology, as well as a decrease in porosity, probably due to the occurrence of side processes. Crosslinking leads to an increase in the sorption capacity for ferrous D-gluconate up to 40%, probably due to the formation of additional hydrogen bonds, making it comparable with HPCD complex formation. The hydrogel obtained by the carbodiimide method has a higher degree of crosslinking, but lower sorption capacity, which indicates the possibility of regulating the properties of the carrier by selecting the crosslinking conditions. Synthetized hydrogels were stable upon storage at room temperature for at least 60 days according to the ATR-FTIR spectroscopy (Figure S4).

Amide crosslinks undergo trypsinolysis, as evidenced by the disappearance of the Amide I and Amide II bands, as well as the CH_2_ stretching bands in the sample spectra after 30 min of incubation with the enzyme. Trypsinolysis occurs over the entire gel surface, as evidenced by the IR microscopy maps for the corresponding absorption bands.

Iron release experiments have confirmed that amide crosslinks significantly slow down premature release in the stomach, while in the intestine, the process is accelerated many times over.

Thus, we demonstrated the possibility of creating a new class of organosilicon hydrogels with trypsin sensitivity for the potential delivery of antianemic drugs. The data obtained open up new prospects for the design of oral dosage forms for improved therapy of iron deficiency anemia.

## 4. Materials and Methods

### 4.1. Materials

Methyltriethoxysilane (Reakhim, Moscow, Russia); 3-aminopropyltriethoxysilane (ABCR, Karlsruhe, Germany); hydrochloric acid (HCl, 35%, 36.46 g/mol, 1.18 g/cm^3^, purity ≥ 99%, Khimmed, Moscow, Russia); anhydrous sodium hydroxide (NaOH, 39.99 g/mol, purity ≥ 95%, Komponent Reaktiv, Moscow, Russia); 2-hydroxypropyl-β-cyclodextrin with degree of substitution 6.0–8.0 ((C_6_H_9_O_5_)_7_(C_3_H_7_O)_n_, 1134.98 + n × 58 g/mol, Zibo Qianhui Biological Technology Co., Zibo, China); Iron (III) chloride hexahydrate (FeCl_3_ × 6H_2_O), 270.30 g/mol, purity 98%, EDC × HCl for coupling, SOCl_2_, DMF, THF, NaHCO_3_, adipic acid, Congo red dye, trypsin, pepsin, KH_2_PO_4_, Na_2_HPO_4_ (Sigma-Aldrich, Saint Louis, MO, USA); ferrous gluconate dihydrate (Fe[HOCH_2_(CHOH)_4_CO_2_]_2_ × 2H_2_O, 99.7%, Ruschim, Moscow, Russia); sodium chloride (NaCl, 58.44 g/mol, purity 99.9%, Reakhim, Moscow, Russia); NH_4_Fe(SO_4_)_2_ × 12H_2_O, NH_2_OH × HCl, ortho-Phenanthroline × 12H_2_O, H_2_SO_4_, NH_4_OH, acetate buffer solution—all (Ximiks, Moscow, Russia), Na_2_B_4_O_7_ × 10H_2_O, KCl (Reakhim, Moscow, Russia).

### 4.2. Methods

#### 4.2.1. Synthesis of (AP/M)SSO Hydrogels

Synthesis of (AP/M)SSO hydrogel PO4 (30% mol. APTEOS). 3-aminopropyltriethoxysilane (48.7 g, 0.06 mol) was added to methyltriethoxysilane (78.4 g, 0.114 mol). Then water (62 mL) was added to a stirred mixture. The reaction mixture was stirred for 30 min to give a colorless dense solution. The substance obtained was left for 20 h for aging and then was rinsed on a filter with water to a neutral reaction (pH = 7). The synthesis was carried out at room temperature. Spectrum NMR ^13^C (400 MHz): −2.3 (C-4); 10.9 (C-1); 27.7 (C-2); 44.9 (C-3). Spectrum NMR ^29^Si (400 MHz): −66.4 (Si-1); −57.4 (Si-2).

Synthesis of (AP/M)SSO hydrogel PO5 (30% mol. APTEOS with addition of ethanol). 3-aminopropyltriethoxysilane (48.7 g, 0.06 mol) was added to methyltriethoxysilane (78.4 g, 0.114 mol). Then ethanol (21 mL) and water (62 mL) were added to a stirred mixture sequentially. The reaction mixture was stirred for 30 min to give a colorless dense solution. The substance obtained was left for 20 h for aging and then was rinsed on a filter with water to a neutral reaction (pH = 7). The synthesis was carried out at room temperature. Spectrum NMR ^13^C (400 MHz): −2.3 (C-4); 10.9 (C-1); 27.7 (C-2); 44.9 (C-3). Spectrum NMR ^29^Si (400 MHz): −66.4 (Si-1); −57.4 (Si-2).

Synthesis of (AP/M)SSO hydrogel PO13 (20% mol. APTEOS with addition of ethanol and alkali). 3-aminopropyltriethoxysilane (7.75 g, 0.035 mol) was added to methyltriethoxysilane (25 g, 0.14 mol). Then ethanol (21 mL) and water solution of NaOH (0.311 g, 41 mL) were added to a stirred mixture sequentially. The reaction mixture was stirred for 30 min to give a colorless dense solution. The substance obtained was left for 20 h for aging and then was rinsed on a filter with water to a neutral reaction (pH = 7). The synthesis was carried out at room temperature. Spectrum NMR ^13^C (400 MHz): −2.3 (C-4); 10.9 (C-1); 27.7 (C-2); 44.9 (C-3). Spectrum NMR ^29^Si (400 MHz): −66.4 (Si-1); −57.4 (Si-2).

Synthesis of (AP/M)SSO hydrogel PO14 (20% mol. APTEOS with addition of ethanol and alkali). 3-aminopropyltriethoxysilane (7.75 g, 0.035 mol) was added to methyltriethoxysilane (25 g, 0.14 mol). Then ethanol (21 mL) and water solution of NaOH (0.09 g, 41 mL) were added to a stirred mixture sequentially. The reaction mixture was stirred for 30 min to give a colorless dense solution. The substance obtained was left for 20 h for aging and then was rinsed on a filter with water to a neutral reaction (pH = 7). The synthesis was carried out at room temperature. Spectrum NMR ^13^C (400 MHz): −2.3 (C-4); 10.9 (C-1); 27.7 (C-2); 44.9 (C-3). Spectrum NMR ^29^Si (400 MHz): −66.4 (Si-1); −57.4 (Si-2).

Synthesis of aminated (AP/M)SSO hydrogel PO4@adipoyl dichloride. Adipic acid (0.6 eq., 2.1 × 10^–4^ mol., 31 mg) was suspended in SOCl_2_ (400 μL). DMF (10 μL, catalytic amount) was added and the resulting mixture was heated to boiling point and stirred at reflux for 2 hrs. Upon cooling, the volatiles were evaporated under reduced pressure and the residue was dissolved in THF (700 μL). The resulting mixture was added dropwise to an ice-bath cooled suspension of hydrogel (1 eq., 3.6 × 10^–4^ mol., 260 mg, thoroughly ground inside reaction medium) and NaHCO_3_ (2 eq., 7.0 × 10^–4^ mol., 59 mg) in a mixture of distilled water (2 mL) and THF (700 μL) and left for 3 h energetic agitation. Then, the solid was separated via centrifugation (8000 rpm, 3 min) and washed with distilled water (5 × 1.5 mL) in the same manner, yielding a pale-yellow gel.

Synthesis of aminated (AP/M)SSO hydrogel PO4@EDC.

Hydrogel (1 eq., 3.6 × 10^–4^ mol. (NH_2_), 260 mg) and adipic acid (0.5 eq., 1.8 × 10^–4^ mol., 26 mg) were agitated vigorously in distilled water (2 mL) until all distinct particles suspended or dissolved (~5 h), followed by the addition of EDCl∙HCl (3 eq., 1.1 × 10^–3^ mol., 207 mg). The resulting mixture was left for overnight stirring, after which the solid was separated via centrifugation (8000 rpm, 3 min) and washed with distilled water (5 × 1.5 mL) in the same manner, yielding a semi-transparent gel.

#### 4.2.2. Determination of the Sorption Capacity of Hydrogels with Congo Red

A standard solution of Congo red (50 mL) was added to 2.0 mL of hydrogel. A mixture was stirred for 30 min, then centrifuged for 20 min at a speed of 6000 rpm. Next, 2 mL of the supernatant liquid was taken and placed in a 50 mL volumetric flask. Then brought the volume to the mark with 0.02 M sodium chloride solution and mix. The optical density of the solution at the absorption maximum at a wavelength of 492 nm was measured by UV spectroscopy using a cuvette with a layer thickness of 10 mm. A 0.02 M sodium chloride solution was used as a reference solution. In parallel, the optical density of the Congo red solution was measured by UV spectroscopy. The adsorption activity of silica gel was calculated by the equation:

$$X=\frac{(A_{0}-A)\times 50000}{A_{0}\times a\times M}$$

A_0_—optical density of the solution of Congo red; A—optical density of the solution of Congo red after sorption; a_0_—Congo red, g; a—hydrogel, g; M—Congo red molecular mass, g/mol.

Preparation of Congo red solution. A solution of sodium chloride (0.02 M, 500 mL) was added to Congo red (0.381 g, 0.0005 mol) placed in a volumetric flask with a capacity of 1000 mL. Then it was diluted to the mark with the same solution and mix. Then 2 mL of Congo red solution was placed in a volumetric flask with a capacity of 50 mL, and the volume was adjusted to the mark with 0.02 M sodium chloride solution and mixed.

#### 4.2.3. Preparing of Iron Containing Solutions

Ferrous D-gluconate solution was prepared as follows: 1.14986 g ferrous D-gluconate with a purity of 0.997 was dissolved in distilled water (10 mL). To accelerate the dissolution, the flask was immersed in hot water and shaken periodically. The concentration of the solution was 0.24 M.

Ferric chloride solution was prepared as follows: 3.30818 g ferric chloride with a purity of 0.98 was dissolved in distilled water (45 mL). The concentration of the solution was 0.27 M.

#### 4.2.4. Iron Sorption by the Hydrogels

Ferrous D-gluconate solution (1 mL, 0.24 M) or ferric chloride solution (1 mL, 0.27 M) was added to the 100 mg of hydrogel. The hydrogels were placed into an incubator for 1.5 h (37 °C, 200 rpm) followed by centrifugation. The supernatants’ iron content was measured using an ortho-phenanthroline assay.

#### 4.2.5. Ortho-Phenanthroline Assay

The method is based on obtaining a colored complex of Fe^2+^ with ortho-phenanthroline and measuring its optical density via UV-VIS spectroscopy [26]. All iron ions are converted into Fe^2+^ by a reduction reaction with hydroxylamine (0.1 g/mL aqueous solution).

In a typical experiment, the aliquot of Fe-containing solution (up to 20 mL) was mixed with NH_2_OH × HCl solution (1 mL), 500 mM acetate buffer solution pH 4.5 (2 mL), orthophenanthroline solution (1.5 mL), and then diluted till 50 mL by distilled water. The colored adduct was detected via UV-VIS spectrometry at 510 nm. The concentration of Fe^2+^ was calculated by means of the calibration curve.

#### 4.2.6. Iron Release Experiments

The release experiment was carried out in simulated gastrointestinal fluids with proteolytic enzymes according to the previously published method [27]. SGF (simulated gastric media) and SIF (simulated intestinal media) were prepared in accordance with US Pharmacopeia (USP).

SGF media was prepared as a Clark–Lubs solution: pH 1.4: 0.053 M HCl, 0.05 M KCl, 0.016 mg/mL pepsin. SIF media was prepared as a sodium–phosphate buffer solution: pH 7.4: 0.07 M KH_2_PO_4_, 0.07 M Na_2_HPO_4_, 0.065 mg/mL trypsin.

Samples of the loaded hydrogel (20 mg) were added to 10 mL of the SGF. After 2 h of incubation at 37 °C, the samples were moved to the SGF for 6 h. Every 20 min, a sample of 2 mL was taken, after which the sample was filled with a fresh portion of the medium. The main pipeline of the experiment is presented in Scheme 4.

#### 4.2.7. Trypsinolysis Assay

Trypsin solution was prepared using borate buffer (0.00115 g, 5 mL of buffer). The active site concentration was 10^−5^ M. Hydrogels (mass nearly 0.02 g) were filled with 0.5 mL of buffer and 0.5 mL of trypsin solution and then placed into the incubator (37 °C, 200 rpm). Samples were taken sequentially after 10, 15, 30, 60, and 90 min and then washed three times with borate buffer followed by three times washing with distilled water. The hydrogels after trypsinolysis were explored using ATR-FTIR spectroscopy and IR microscopy.

#### 4.2.8. Instrumental Analysis

The ^29^Si NMR spectra of (AP/M)SSO hydrogels were registered on a Bruker Avance III 400 spectrometer (Bruker, Elltingen, Germany). The analysis was carried out using a solid-state sensor with rotation at a magic angle with a frequency of 8 kHz using cross-polarization and decoupling from 1 H.

The ^13^C NMR spectra of (AP/M)SSO hydrogels were registered on a Bruker Avance III 400 spectrometer. The analysis was carried out using a solid-state sensor with rotation at a magic angle with a frequency of 12 kHz using cross-polarization and decoupling from 1 H.

The UV-spectra were recorded with an Ultrospec 2100 pro instrument (Amersham Biosciences, Amersham, UK) within a wavelength range of 200–700 nm in a 1 mL quartz cell (Hellma Analytics, Müllheim, Germany).

The FTIR-spectra were recorded using a Tensor 27 Fourier-transform IR spectrometer (Bruker, Elltingen, Germany) equipped with an MCT detector cooled with liquid nitrogen and a thermostat (Huber, Offenburg, Germany). The measurements were carried out in a thermostated BioATR II cell with an attenuated total internal reflection (ATR, Bruker, Germany) using a single-reflection ZnSe crystal at 22 °C and a constant purge rate of the system with dry air using a Jun-Air apparatus (Jun-Air, Redditch, UK). An aliquot portion of the sample (40 μL) was applied to the crystal of the ATR cell; the spectrum was recorded thrice in the range from 4000 to 950 cm^−1^ with a resolution of 1 cm^−1^; it was scanned 70 times and averaged. The background signal was recorded similarly. The Opus 7.0 software was used to analyze the spectra.

FTIR microscopy studies with mapping of main peak intensities maps were recorded using the MICRAN-3 FTIR microscope (Simex, Novosibirsk, Russia) equipped with a liquid nitrogen-cooled MCT detector.

N_2_ adsorption–desorption isotherms were measured on the TMAX 3H-2000PM2 gas sorption analyzer (Xiamen Tmax Battery Equipments Limited, Xiamen, China) at −196 °C. All samples were preliminarily outgassed under vacuum at 120 °C for 12 h. The Brunauer–Emmett–Teller (BET) specific surface area (SSA) was determined from the linear plot in the relative pressure range of 0.05–0.3 P/P0. The pore size distribution was calculated in the relative pressure range (P/P0) from 0.1 to 0.99 using the Barrett−Joyner−Halenda (BJH) method. Only the desorption branch of the isotherm was used for the analysis.

Scanning electron microscopy (SEM) was performed using an FEI Helios G4 CX dual beam scanning electron microscope (Hillsboro, OR, USA) with SPI and TEAM 3D IQ 3.11 software. Preliminarily, a thin layer of Ag (10 nm) was coated to samples using SPI Supplies (West Chester, PA, USA).

Elemental analysis: Carbon and hydrogen elemental analyses were carried out by express gravimetric analysis. The method is based on the pyrolytic combustion of a 3–5 mg sample of the substance in a platinum boat in a quartz tube in an oxygen flow at 1000 °C in the presence of PbO. The sample combustion results in the quantitative formation of CO_2_ and H_2_O which are absorbed outside the combustion tube by ascarite and anhydrone, respectively. The C and H contents were calculated based on the weight gain of the absorption apparatus. The method error is 0.30–0.50%. The silicon content was determined spectrophotometrically on a Cary-100 instrument in the form of a silicon molybdenum blue complex. The method error is 0.30–0.50%. Nitrogen elemental analysis was carried out by the Dumas–Preglia–Korshun method. A suspension of the substance (3–10 mg) was incinerated in an atmosphere of carbon dioxide in a quartz container filled with oxidizer. The container with the sample was placed in a combustion tube and combustion was carried out in an atmosphere of CO_2_. Under these conditions, nitrogen from the substance is converted into elemental nitrogen, which is displaced by a current of carbon dioxide into the nitrogen meter and determined volumetrically. The method error is 0.30–0.50%.

## Figures and Tables

**Table 1:** Structural–functional properties of (AP/M)SSO hydrogels.

Sample	APTES, Molar %	NaOH, g	EtOH, mL	Sorption Capacity, μmole/g *	S (BET), m^2^/g	D90, nm	Pore Volume, mL/g
PO4	30	0	0	84.9 ± 4.3	210	6.1	0.36
PO5	30	0	10	82.0 ± 3.3	19	5.2	0.03
PO13	20	0.27	21	43.4 ± 1.8	328	6.6	0.61
PO14	20	0.09	21	47.0 ± 1.9	266	11.3	0.73
PO4@EDC					251	4.8	0.38
PO4@adipoyl dichloride					15	18.8	0.01

* Congo red test.

**Table 2:** Elemental analysis data for (AP/M)SSO hydrogels.

Sample	% C	% H	% N	% Si
PO4	23.73	5.69	5.54	34.71
PO5	23.77	5.63	5.44	34.58
PO13	21.86	5.58	3.35	36.69
PO14	21.84	5.54	3.30	36.72

**Table 3:** Efficiency of iron salt sorption in (AP/M)SSO hydrogels (calculated as Fe weight). SD, n = 3. Aqueous media, pH 7.0, 22 °C.

Sample	FeCl_3_, %	Ferrous D-Gluconate, %	Ferrous D-Gluconate@HPCD, %
PO4	63 ± 4	11 ± 6	35 ± 9
PO5	53 ± 7	13 ± 8	41 ± 8
PO13	60 ± 15	<5	-
PO14	69 ± 12	<5	-
PO4@adipoyl dichloride	-	55 ± 8	-
PO4@EDC	-	28 ± 8	-

## Data Availability

The original contributions presented in this study are included in the article/Supplementary Material. Further inquiries can be directed to the corresponding author.

## Supplementary Materials

The following supporting information can be downloaded at: https://www.mdpi.com/article/10.3390/gels11020118/s1, Figure S1: Typical ^13^C and ^29^Si NMR spectra for (AP/M)SSO hydrogels PO4 and PO13; Figure S2: N_2_ isotherm of sorption—desorption for (AP/M)SSO hydrogels PO4, PO5, PO13, PO14 as well as PO4@EDC and PO4@adipoyl dichloride; Figure S3: ATR-FTIR-spectra of 0.1 M HPCD (black line), 0.24 M ferrous D-gluconate (green line) and complex ferrous D-gluconate-HPCD in 1:1 (red line).pH 4.0 (0.1 mM HCl), 22 °C. Figure S4. ATR-FTIR-spectra of 0PO4, PO4@EDC, and PO4@adipoyl dichloride hydrogels before and after 60 days of storage at room temperature.
