# Supplementary material for: Amidated and Aminated PMSSO-Hydrogels as a Promising Enzyme-Sensitive Vehicle for Antianemic Drugs
Source: Gels. 2025 Feb 6;11(2):118. doi: 10.3390/gels11020118 (PMC11854879; doi:10.3390/gels11020118)
Supplement: Supplementary file 1 [file gels-11-00118-s001.zip › gels-3423826-supplementary.pdf]

Supplementary material for the manuscript

Amidated and Aminated PMSSO-Hydrogels as a Promising Enzyme-Sensitive Vehicle for Antianemic Drugs

Polina Orlova [1], Ivan Meshkov [2], Sergei Sharikov [1], Vsevolod Frolov [1], Anna Skuredina [1], Pavel Markov [1], Zoya Bobyleva [1], Grigorii Lakienko [1], Egor Latipov [3], Ilya Kolmogorov [1], Sergey Vasiliev [4], Alexandra Kalinina [2], Aziz Muzafarov [2] and Irina Le-Deygen [1,*]

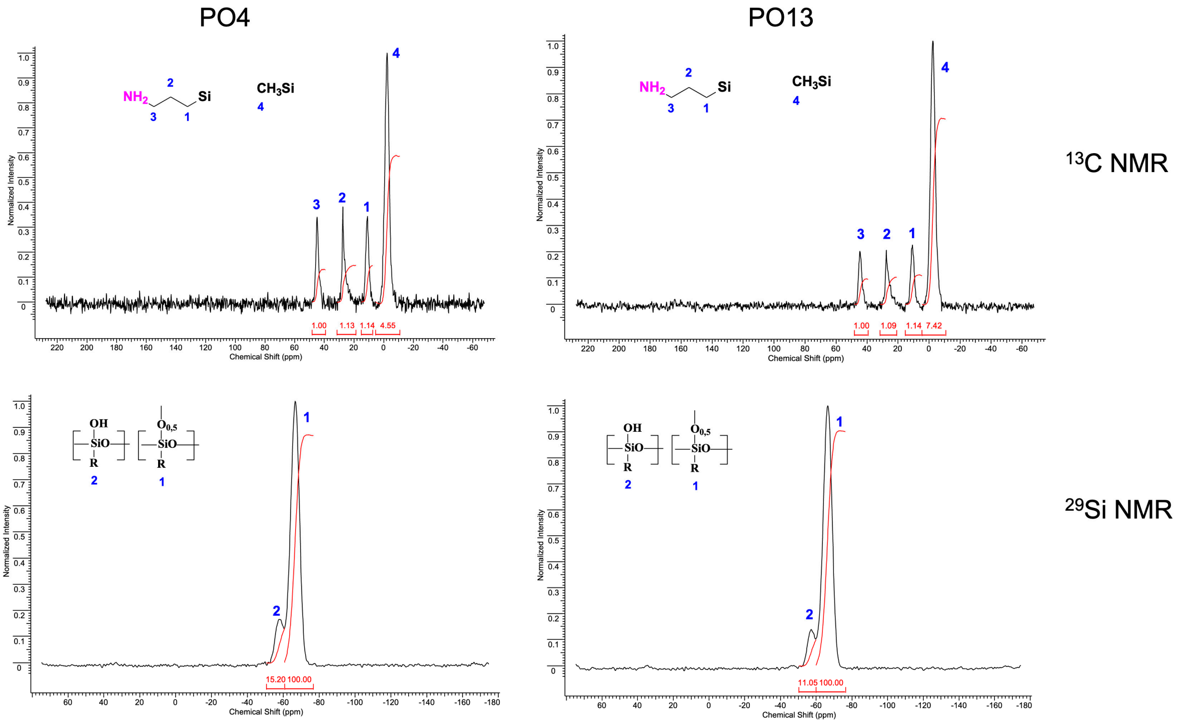

Figure S1. Typical ^{13}C and ^{29}Si NMR spectra for (AP/M)SSO hydrogels PO4 and PO13.

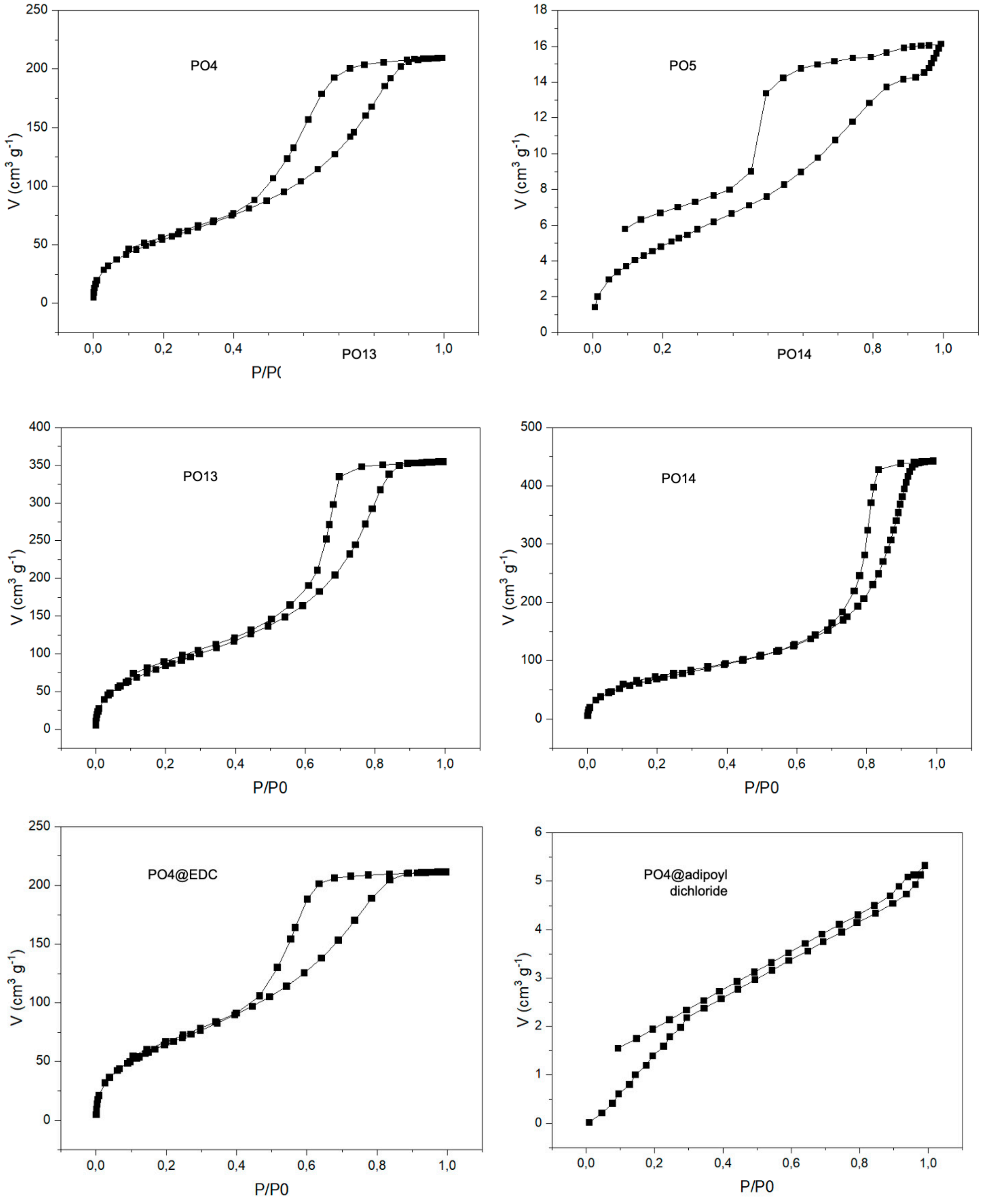

Figure S2. N_2 isotherm of sorption – desorption for (AP/M)SSO hydrogels PO4, PO5, PO13, PO14 as well as PO4@EDC and PO4@adipoyl dichloride.

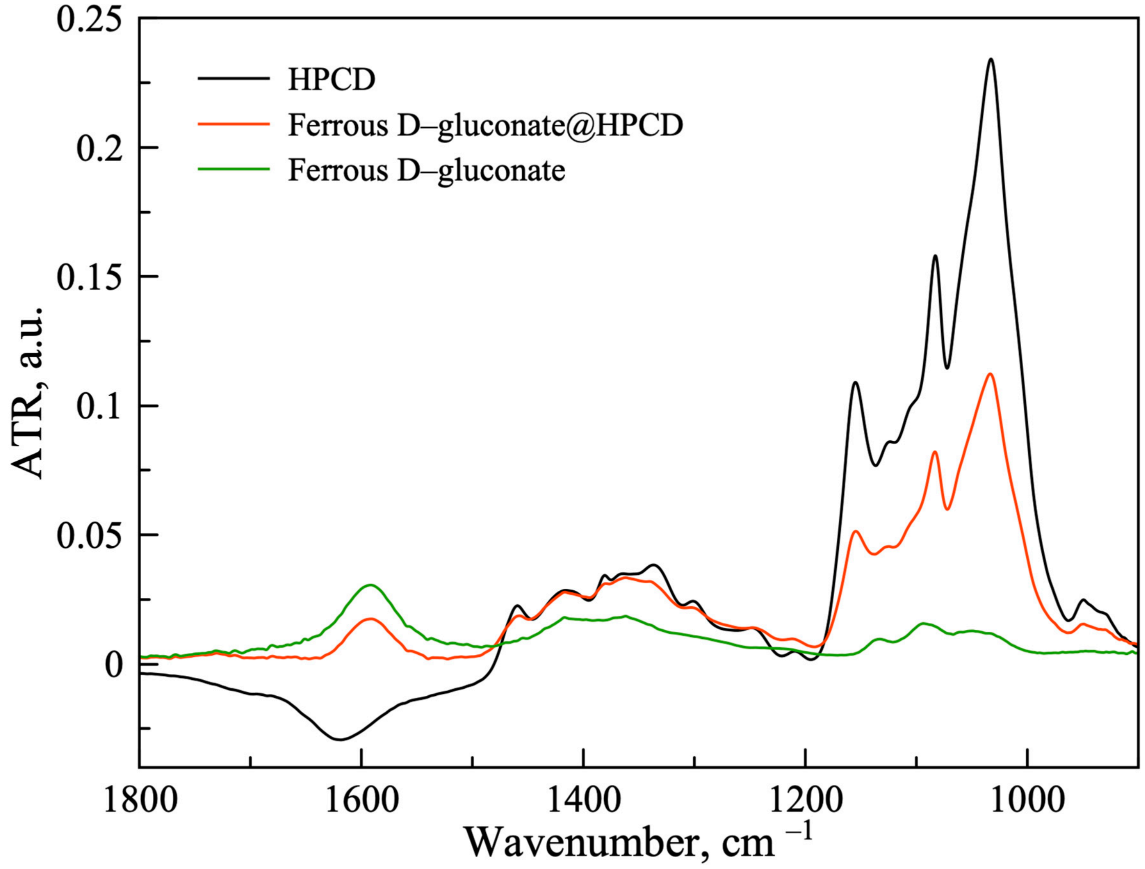

Figure S3. ATR-FTIR-spectra of 0.1 M HPCD (black line), 0.24 M ferrous D-gluconate (green line) and complex ferrous D-gluconate-HPCD in 1:1 (red line).pH 4.0 (0.1 mM HCl), 22 °C.

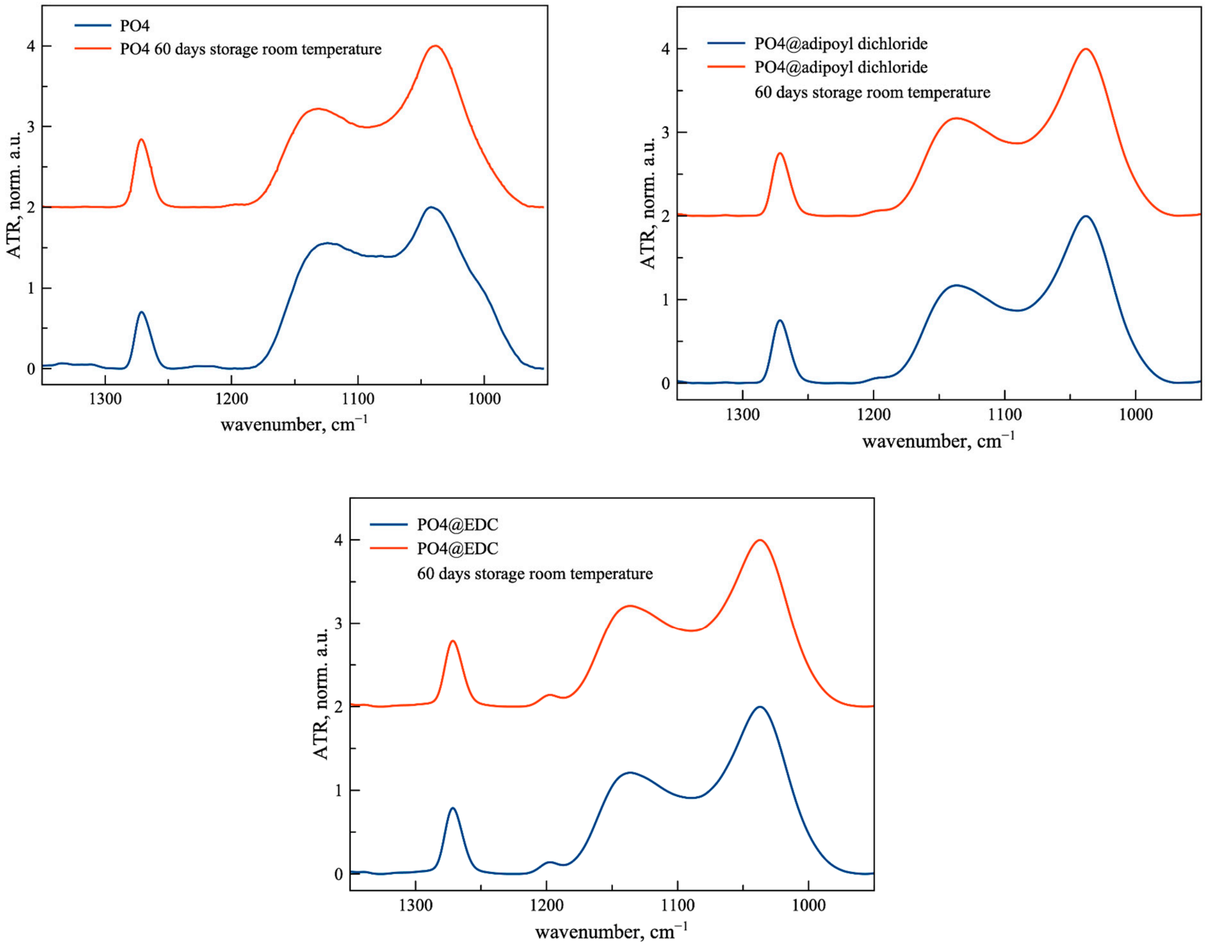

Figure S4. ATR-FTIR-spectra of 0PO4, PO4@EDC and PO4@adipoyl dichloride hydrogels before and after 60 days of storage at room temperature.
